# Different keratoconus definitions can lead to substantial prevalence disparities in population-based studies

**DOI:** 10.1038/s41598-025-87532-4

**Published:** 2025-01-28

**Authors:** Hasan Shabani, Bart T. H. van Dooren, Magda A. Meester-Smoor, Annette A. J. M. Geerards, Caroline C. W. Klaver, Wishal D. Ramdas

**Affiliations:** 1 Department of Ophthalmology, Erasmus Medical Centre, Rotterdam, The Netherlands; 2 Department of Epidemiology, Erasmus Medical Centre, Rotterdam, The Netherlands; 3 Cornea Centre, The Rotterdam Eye Hospital, Rotterdam, The Netherlands; 4 Department of Ophthalmology, Amphia Hospital, Breda, The Netherlands; 5 Department of Ophthalmology, Radboud University Medical Centre, Nijmegen, The Netherlands; 6 Institute of Molecular and Clinical Ophthalmology, University of Basel, Basel, Switzerland

**Keywords:** Epidemiology, Corneal diseases, Vision disorders

## Abstract

This report explores the prevalence of keratoconus in a population-based cohort of adults aged 40 or older according to ten different definitions. All Rotterdam Study participants with reliable Pentacam scans and no prior corneal refractive surgery were cross-sectionally analysed (*n* = 2660). First, we applied a novel evidence-based definition. Suspected keratoconus was defined as having at least one eye with a final D-index (BAD-D) ≥ 2.6. Manifest keratoconus was defined as having at least one eye with: (1) BAD-D ≥ 2.6; and (2) a score of at least 4/10 on the novel Rotterdam Keratoconus Scale (RKS); and (3) a confirming assessment of the relevant Pentacam maps; and (4) meeting Holladay’s criteria in case of recent contact lens usage. Using this proposed definition, 72 participants (2.71%, 95%CI: 2.16–3.40%) had suspected keratoconus, while 10 participants (0.38%, 95%CI: 0.20–0.69%) had manifest keratoconus. To assess reproducibility, two specialists independently applied the proposed definition, with a substantial inter-observer agreement (Kappa = 0.74). Interestingly, 6(60%) patients were unaware of having keratoconus. Applying nine alternative definitions from similar screening studies produced prevalence estimates ranging from 0.19 to 9.29% in the same cohort. Moreover, counting solely on a BAD-D cutoff of 2.6 to define keratoconus was unreliable, with a low positive predictive value of 14%. These findings explain partially the large heterogeneity in the reported keratoconus prevalences, underscoring the need for a standardized definition.

**Supplementary Information:**

The online version contains supplementary material available at 10.1038/s41598-025-87532-4.

## Introduction

Keratoconus is a progressive eye disease that affects the shape and transparency of the cornea. If left untreated, this disease can lead to distorted vision, visual impairment and in rare cases, blindness. Keratoconus usually begins around puberty, causing patients to have a significantly impaired vision-related quality of life, which declines further over time^1^. Additionally, keratoconus is still one of the most common indications for keratoplasty in some parts of the world^2,3^.

The reported prevalence of keratoconus varies widely across studies, ranging from as low as 0.0002% in the Urals in Russia^4^ to as high as 4.79% amongst Saudi adolescents in Riyadh^5^. Understanding the real prevalence of this condition is important from a public health perspective, as it can inform resource allocation decisions and future screening programs. However, many prior studies have been limited by selection biases, variability in the used diagnostic criteria and discrepancies in access to ophthalmic care. This makes comparisons across studies challenging^6^.

Furthermore, a sizable percentage of keratoconus patients may be unaware of having the disease^7–11^. This suggests that studies using hospital or insurance data may underestimate the true prevalence, as they primarily capture patients at more advanced stages. In contrast, large population-based screening studies have the potential to capture the full spectrum of keratoconus. When appropriately designed, these studies are less prone to methodological biases and less likely to be affected by low unawareness or variations in referral patterns.

We aim therefore to describe the prevalence of keratoconus cases in a population-based setting according to a new tomography-based definition that can be easily applied in epidemiological research. Additionally, we will review other published epidemiological definitions from the literature and evaluate their comparability when applied to the same population-based dataset.

## Methods

### Population

The Rotterdam Study is a large population-based cohort study that began in 1990 with the aim of investigating determinants of (chronic) diseases in the elderly. The study population comprises 17,931 participants aged 40 years or older who reside in the district of Ommoord in Rotterdam, the Netherlands. The study design has been described in detail elsewhere^12^. Our analysis encompassed all Rotterdam Study participants who underwent at least one Scheimpflug imaging scan of acceptable quality and had no history of corneal refractive surgery.

### Data collection

All consenting participants underwent extensive ophthalmic examinations, including Scheimpflug tomography (December 2016-onwards). This was done using Pentacam HR (Oculus Optikgeräte GmbH, Wetzlar, Germany). Eligible participants were identified by filtering all Pentacam scans available at The Rotterdam Study. Only reliable scans were considered. Reliability was assessed based on having an “OK” quality score as given by the Pentacam. In case of multiple reliable scans made on the same visit, the last reliable scan was analysed. More information about the scan capture and export protocols can be found in Supplementary Methods S1. Participants also underwent ocular biometry of both eyes using Lenstar 900 Optical Biometer (Haag-Streit, Koeniz, Switzerland). Lenstar data was used in our study to report the axial length among participants, and to apply an alternative (Lenstar based) definition from the literature. The best corrected visual acuity (BCVA) was determined using a phoropter. We note that this method does not include correction with rigid gas-permeable or scleral lenses, which are often used in keratoconus cases to achieve maximal visual correction.

In addition to ophthalmic testing, demographic data and ocular and medical history were collected during interviews carried out by trained research assistants. Educational attainment was assessed by asking participants whether they have obtained a university (or a higher vocational training) diploma or not. Ethnicity was inferred from principal components analysis of the participant’s genetic array data using Admixture (v1.3.0)^13^.

### Proposed definition

The final D of the Belin-Ambrosio enhanced ectasia display (BAD-D) was used for initial screening. We defined suspected (i.e., probable) keratoconus as having a reading of ≥ 2.6 in at least one eye, with no history of corneal refractive surgery.

Manifest (i.e., definite) keratoconus was defined as having at least one eye that meets all of the following 4 criteria: **(1)** BAD-D ≥ 2.6; and **(2)** a score of ≥ 4/10 on the novel Rotterdam Keratoconus Scale (RKS, Table 1); and **(3)** a confirming assessment of the relevant Pentacam displays; and **(4)** a positive assessment according to Holladay’s criteria in case of contact lens usage in the last two weeks^14^. The qualitative elements (criteria 2, 3 and 4) of this definition were independently assessed by two corneal specialists (A.G. and B.v.D.). Agreement rates were assessed, and in case of disagreement, consensus was reached through discussion.

**Table 1:** The 10-points Rotterdam Keratoconus Scale (RKS).

1- Significantly deviating (≥ 2 SDs) anterior or posterior elevation at the thinnest point:	
In myopic eyes ≥ 5.7 μm anterior elevation or ≥ 13 μm posterior elevation	
In Hyperopic eyes ≥ 4.3 μm anterior elevation or ≥ 22.1 posteriorly.	
2- Significant (≥ 2 SDs) anterior elevation difference (Df).	
3- Significant (≥ 2 SDs) posterior elevation difference (Db).	
4- Significantly deviating (≥ 2 SDs) average progression index (Dp).	
5- Significantly deviating (≥ 2 SDs) maximum Ambrosio’s relational thickness (Da).	
6- Significantly deviating (≥ 2 SDs) thinnest corneal pachymetry (Dt).	
7- Inferior-superior power asymmetry of 1.6 D or more.	
8- Mean keratometry of 47.3 D or higher.	
9- Skewed radial axes with an angle above 21 degrees.	
10- Significant topographic or pachymetric asymmetry between the right and left eyes.	

The chosen cutoffs for the first nine parameters represent around two standard deviations from the mean in a healthy population. Assessment of the tenth parameter was left to the discretion of the specialist. A score of 4 or more in at least one eye was one of the requirements for a case to be defined as manifest (i.e., definite) keratoconus. SD, standard deviations.

### Proposed definition: rationale

BAD-D is a multivariable index that is calculated through linear regression of nine key corneal variables^15^. It has been reported to have an excellent sensitivity (91–100%) and an excellent specificity (91–100%) for detecting keratoconus^16^. A cutoff of 2.6 is frequently used to diagnose or screen for keratoconus^17–19^. Some experts suggested that a higher BAD-D cutoff might be more appropriate for older people^20^. Due to the lack of published data supporting this, we opted for the traditional cutoff of 2.6.

Despite its numerous advantages, BAD-D was not designed or intended to be a standalone diagnostic index^21^. Therefore, to ensure a comprehensive diagnostic approach, we incorporated the novel RKS scale in the definition (Table 1). This scale is designed to quantify and standardize the diagnostic process. It includes 10 key clinical signs of keratoconus, drawn from the literature^22–28^. The diversity of signs which comprise the RKS aims to capture the phenotypic diversity of keratoconus. Nine of the ten included signs had clear cutoffs, established at about two standard deviations (≥ 95th percentile) away from the mean in a healthy population^23–26^. The last sign (interocular asymmetry) was left to the discretion of the specialist, making it a subjective element. The RKS systematically evaluates three signs of abnormal corneal elevation, three signs of abnormal pachymetry (distribution), three signs of abnormal curvature (distribution), and one sign of interocular asymmetry. This systematic approach ensures that four key corneal domains are assessed. A minimum score of 4/10 was found to be appropriate for distinguishing manifest from subclinical cases. This threshold reflects the clinical judgment and experience of the specialists who compiled the scale, aiming to balance sensitivity and specificity in the diagnosis. The suggested threshold also ensures the presence of abnormalities in at least two of the four assessed corneal domains.

In addition to the RKS, we incorporated a qualitative review of the topometric, refractive and enhanced ectasia Pentacam displays carried out by specialists to ensure that the maps are consistent with keratoconus. The main focus of this step was excluding false positives which might arise for instance due to the presence of other ectasias, ocular alignment issues, scan errors not detected by Pentacam, and/or unreported previous corneal surgery. Holladay’s criteria were also examined when contact lens warpage could confound the diagnosis^14^.

### Alternative definitions: literature review and comparative analysis

We quickly reviewed the peer-reviewed literature to find other relevant population-based screening studies. The published keratoconus definitions were summarized, and the definitions that were readily applicable in our cohort were tested and compared. Our aim was to quantify the variability in prevalence figures arising from the use of different diagnostic criteria. A description of the performed literature review is available in Supplementary Methods S2, and the results are summarized in Supplementary Tables S1 and S2. In short, 27 relevant studies were identified^5,7–11,18,19,29–47^. The variety of diagnostic instruments used across studies poses a challenge, as diagnostic indices from different platforms are often not interchangeable. This limitation prevented us from applying definitions that rely on device-specific indices. A total of nine keratoconus definitions were readily applicable to our dataset (Supplementary Table S2). Amongst those definitions, seven relied on the Pentacam, one on the Lenstar, and one on a manual keratometer (Appassawamy Assoc, Chennai, India). This last definition was applied to the Rotterdam Study data using comparable Pentacam data transformed with a suitable corneal refractive index of 1.3375^29^.

Each readily applicable definition was applied to the Rotterdam Study dataset. We reported and compared the prevalence figures according to each definition. We also assessed the inter-observer agreement levels between these definitions.

### Reliability of a high BAD-D reading

Multiple recent studies relied on BAD-D as a quick standalone diagnostic index for keratoconus^40,44,48–50^. To assess the validity of this approach, we calculated the positive predictive value (PPV) of BAD-D when used for this purpose. Our extensive proposed definition was used as the ground truth for this analysis. PPV was calculated using a traditional BAD-D cutoff of 2.6, and another cutoff of 3.4. The latter was selected as the optimal cutoff based on Youden’s index J value. This method defines the best cutoff as the point maximizing the difference between true positive rate and false positive rate over all possible cut-point values.

### Risk factors analysis

The investigated risk factors for keratoconus included xerosis cutis (dry skin), itchy skin disorders, asthma, dust mite allergy, hay fever, diabetes, smoking, and snoring. Data on other relevant risk factors such as eye rubbing, or a family history of keratoconus are not routinely collected in The Rotterdam Study. More details about the interview questions and the data collection procedure are available in Supplementary Methods S3.

### Statistical analyses

Given that the proposed method to define definite keratoconus included a subjective part, we calculated the inter-observer agreement percentage and Cohen’s kappa. We also analysed differences in general and ocular characteristics between participants with and without keratoconus. This was done using Mann-Whitney or chi-square tests as appropriate. To compare ocular features, readings were taken from the most severely affected eye of (probable) keratoconus cases and the right eye of healthy participants. When these were missing or unreliable, the other eye readings were used instead. Also, if data from the most recent interview were not available, data from previous interviews were used.

For the comparative analysis, we calculated Cohen’s kappa to assess the agreement levels between the various definitions. We also assessed the suspected risk factors individually under each definition using multivariable logistic regression adjusted for age and sex. Next, we calculated the odds ratio (OR) with 95% confidence intervals (CI) for each potential risk factor. Pairwise deletion was applied to handle missing values. Statistical tests were carried out using SPSS v29.0 for Windows (SPSS Inc, Armonk, NY, USA)^51^, R v4.1.1 (R Foundation for Statistical Computing, Vienna, Austria)^52^, and RStudio v1.4.1717 (R Studio, Boston, MA, USA)^53^.

### Ethics approval

The Rotterdam Study has been approved by the Medical Ethics Committee of the Erasmus MC (registration number MEC 02.1015) and by the Dutch Ministry of Health, Welfare and Sport (Population Screening Act WBO, license number 1071272-159521-PG). All participants provided written informed consent to participate in the study and to have their information obtained from treating physicians. The study was conducted according to the provisions of the Declaration of Helsinki.

## Results

### Study population

A total of 2970 participants underwent Pentacam scans. Of these, 2660 participants (90%; mean age ± standard deviation: 59 ± 12 years, 55% females) underwent at least one Scheimpflug scan of adequate quality and had no history of corneal refractive surgery.

### Proposed definition: prevalence, agreement and awareness levels

**Fig. 1:**
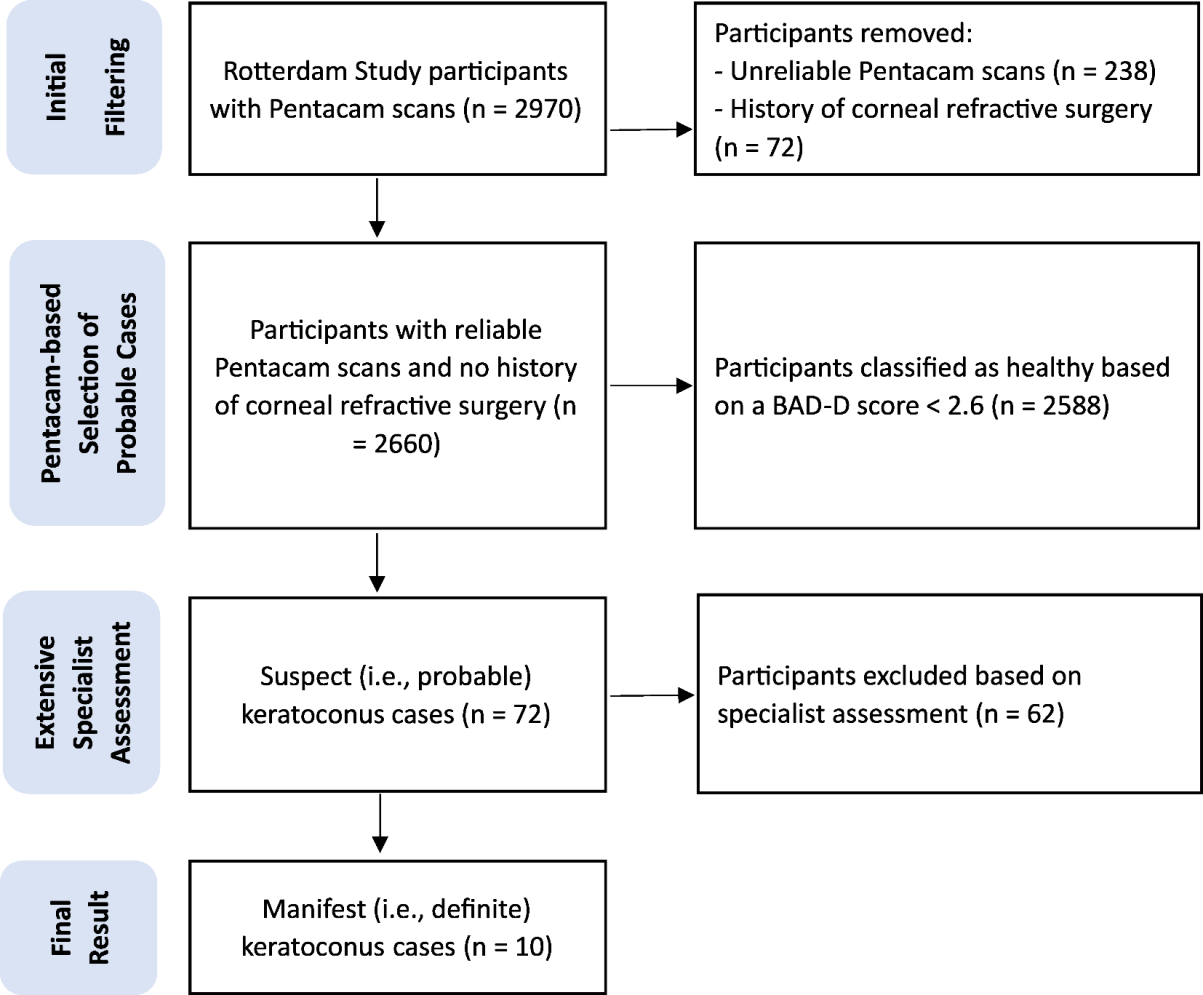
Filtering process according to the proposed keratoconus definition.

Figure 1 illustrates the steps taken to select probable keratoconus cases according to the proposed definition. The prevalence of probable keratoconus was 2.71% (72/2660, 95%CI: 2.16–3.40%), and the prevalence of manifest (i.e., definite) keratoconus was 0.38% (10/2660, 95%CI: 0.20–0.69%). Among the subgroups, the prevalence of manifest keratoconus was 0.34% (8/2380) in Europeans, 0.59% (7/1186) in males and 0.20% (3/1474) in females. The difference in prevalence between males and females was not statistically significant (*P* = 0.11). Table 2 summarizes the characteristics of study participants and the confirmed keratoconus cases.

**Table 2:** General and ocular characteristics of the study groups. Results are presented as mean ± standard deviation unless stated otherwise.

	Missing values, *n*(%)	Keratoconus patients (*n* = 10)	Controls (*n* = 2650)	*P* value
Age, years	-	55.1 ± 8.9	59.4 ± 11.6	0.257^*^
Female, n(%)	-	3 (30)	1471 (55.5)	0.105^†^
Ethnicity, n(%)	126 (4.7)			
Caucasian ancestry, n(%)		8 (80)	2372 (94)	0.065^†^
African ancestry, n(%)		1 (10)	64 (2.5)	0.136^†^
East-Asian ancestry, n(%)		0 (0)	50 (2)	0.653^†^
Admixed ancestry, n(%)		1 (10)	38 (1.5)	**0.029** ^†^
Higher education degree, n(%)	12 (0.5)	2 (20)	851 (32.3)	0.408^†^
Spherical refraction (Diopters)	13 (0.5)	-1.50 ± 3.35	0.04 ± 2.55	0.075^*^
Negative cylinder (Diopters)	262 (9.8)	3.53 ± 1.93	0.88 ± 0.77	**< 0.001** ^*^
Central corneal thickness (micrometers)	0 (0)	487.10 ± 40.01	564.96 ± 37.25	**< 0.001** ^*^
Axial length (millimeters)	401 (15.1)	23.91 ± 1.47	23.77 ± 1.27	0.651^*^
Intra-ocular pressure (mm HG)	68 (2.6)	13.17 ± 3.34	12.89 ± 2.88	0.443^*^

P values in bold are statistically significant at an alpha of 0.05.

^*^ Mann-Whitney U test. ^†^ Chi-square test.

On independent assessment of the 72 probable keratoconus cases, both judges agreed to exclude 58 cases and to include 9 cases. The other 5 cases were initially disputed but consensus was eventually reached. The agreement percentage between the two raters was 93.06% and Cohen’s kappa was 0.74, reflecting substantial agreement between the raters in assessing probable keratoconus cases^54^.

Checking ophthalmic history records of the confirmed keratoconus cases revealed that 6(60%) patients were unaware of their diagnosis or did not report it. Four of the six unaware patients had varying degrees of visual acuity loss. One patient had a BCVA (best corrected visual acuity, measured with a phoropter) of 0.05, two patients had a BCVA of 0.5 and one had a BCVA of 0.63, all measured in the most affected eye of each patient. Bilateral BCVA data is presented in Supplementary Table S4.

### Alternative definitions: comparative prevalence analysis

**Fig. 2:**
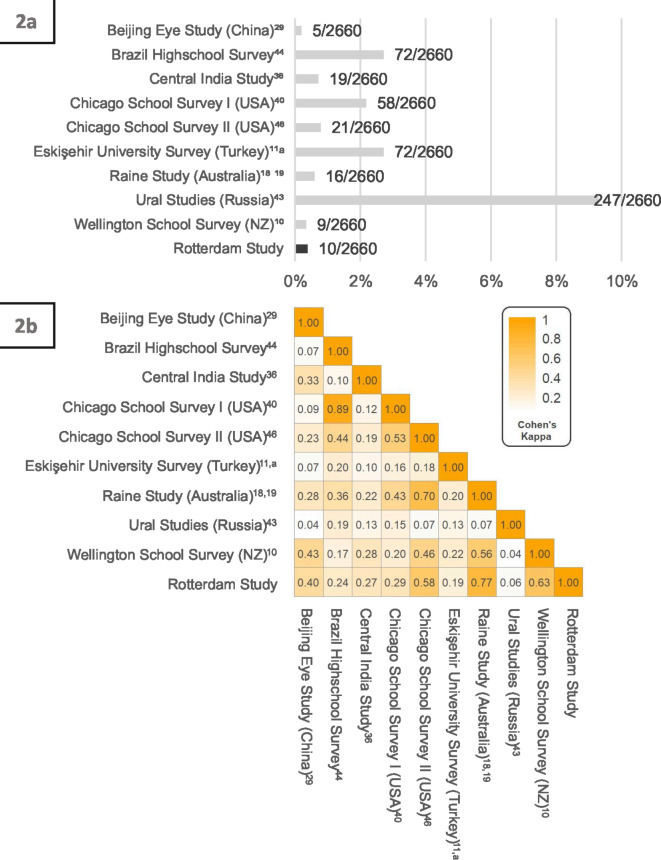
Keratoconus prevalence in The Rotterdam Study according to other published definitions (2a), and their respective agreement levels (2b). ^a^ The study lists two keratoconus definitions. However, Only the definition based on the Topographic Keratoconus index (TKC) was readily applicable to our dataset. The heatmap was generated with the “pheatmap” package in R. All the reported kappa coefficients were statistically significant (*P* < 0.001). NZ, New Zealand.

When applied to the Rotterdam Study dataset, the nine alternative definitions yielded prevalence estimates ranging between 0.19% and 9.29% (Fig. 2a and Supplementary Fig. S1). The definitions based solely on keratometry yielded the two most extreme prevalence figures (0.19% and 9.29%). Similarly, the definitions based solely on BAD-D yielded high prevalence figures (2.18% and 2.71%). Generally, the agreement levels between the applied definition pairs were low, with a median agreement kappa of 0.20. Figure 2b represents an agreement heatmap illustrating the agreement levels among all the compared definitions.

### Positive predictive value of BAD-D

We also evaluated the PPV of BAD-D as a standalone diagnostic index of keratoconus. This was 14% at the frequently used cutoff of 2.6. In other words, only 14% of participants with a BAD-D ≥ 2.6 had manifest keratoconus according to the proposed definition. Using an optimal cutoff of 3.4 improved the PPV slightly to 34%.

### Risk factors analysis

Supplementary Table S3 presents the results of the risk factor analysis. Three of the compared definitions showed a nominally significant association between dry skin and keratoconus (*P* = 0.022, *P* = 0.012 and *P* = 0.004), with the remaining definitions showing concordant effect direction. The analysis also suggested a possible link between hay fever and keratoconus, with all definitions indicating a positive association, one of which nominally significant (*P* = 0.038).

## Discussion

This study offers a comprehensive examination of keratoconus prevalence, highlighting the critical role of diagnostic criteria in shaping keratoconus-related epidemiological findings. Given the plethora of definitions used to diagnose keratoconus in screening studies, we reported the prevalence according to ten different definitions. Interestingly, the prevalence of keratoconus seemed to vary greatly as a function of the applied definition. In the same cohort, the prevalence rate ranged from 0.19 to 9.29%. By comparing the performance of these different definitions, some useful reflections can be made about the real prevalence of keratoconus and how (not) to measure it.

### The proposed definition

If this study had relied solely on elevated maximum keratometry readings or BAD-D levels, it could have made headlines with an unprecedentedly high prevalence of keratoconus in Europe. We preferred instead to propose and rely on a more nuanced and evidence-based definition. This approach may serve as a framework for future clinical and epidemiological research, or at least spark a discussion about the importance of standardizing definitions within the field.

### Keratoconus as a spectrum

The corneal deformation caused by keratoconus exists on a spectrum, and the compared definitions seemed to capture very different degrees of severity on the keratoconus severity spectrum. Therefore, the most important lesson that can be learned from our comparative analysis is that it is really challenging, if not improper, to compare keratoconus prevalence figures based on different epidemiological definitions.

### Keratometry based definitions

Three of the compared definitions were solely based on keratometry readings. Notably, the definition based on maximum keratometry gave the most extreme prevalence figure of 9.3% in the Rotterdam Study. A potential shortcoming of such an approach is that (maximum) keratometry seems to physiologically increase with age during adulthood^55^. This is clearly illustrated in Supplementary Fig. S1 which compares the age-related performance of each definition. We also know nowadays that keratoconus is not interchangeable with corneal steepening, with evidence accumulating about the existence of keratoconus cases with relatively low keratometry^56^. It is therefore not recommended to rely solely on keratometry values.

### BAD-D based definitions

Some other studies relied solely on BAD-D readings to detect keratoconus. However, BAD-D readings were never intended to function as a standalone diagnostic index for keratoconus. This can cause false positives, as Doctor Belin himself (after whom the index is named) previously noted^21^. In our cohort, BAD-D showed an alarmingly low PPV regardless of the used cutoff, underscoring thus the need to combine BAD-D with other signs. It is essential to note that in the case of rare diseases, a diagnostic test can have a low PPV despite having a high sensitivity and specificity. This is sometimes referred to as the false positive paradox. The low PPV of BAD-D might have broader clinical implications. For example, relying solely on it to select refractive surgery patients might be too restrictive, while using it as a standalone indication for early crosslinking (before other signs appear) risks overtreatment. However, the relatively older age of our cohort prevents us from drawing any definitive conclusions on these matters.

### The real prevalence of Keratoconus in Europe

Based on screening, the prevalence of keratoconus in Europe was estimated at 0.38% in our study, 0.49% in Germany, 0.55% in Poland and 0.75% among male military recruits in France^35,41,42^. These numbers seem notably higher than other European studies based on hospital or healthcare registries (0.04% in Denmark, 0.03% in Finland, 0.19% in Norway and 0.27% in The Netherlands)^57–60^. Due to differences in design and setting, the reported differences do not necessarily reflect true underlying prevalence differences between European countries.

### Some geographic prevalence differences are likely real

While definition and design differences matter, they do not seem to explain all the worldwide differences in the reported keratoconus prevalence. For example, the prevalence of Keratoconus in The Raine Study in Australia was recently estimated at a substantial 3.4% among young adults^19^. The Raine Study definition showed a reasonable agreement with our definition. Moreover, both studies were done in a predominantly Caucasian, population-based screening context. However, the prevalence in the Rotterdam Study came out a few folds lower than the Australian Study. It is not clear whether this difference is due to genetics, weather conditions or other factors. Theoretically, an older cohort like the Rotterdam Study is expected to have a higher point prevalence than the younger Raine Study cohort^61^. However, the opposite seems to be the case. A possible explanation for this is that the prevalence of keratoconus might be on the rise in younger generations.

### Unaware patients

**Fig. 3:**
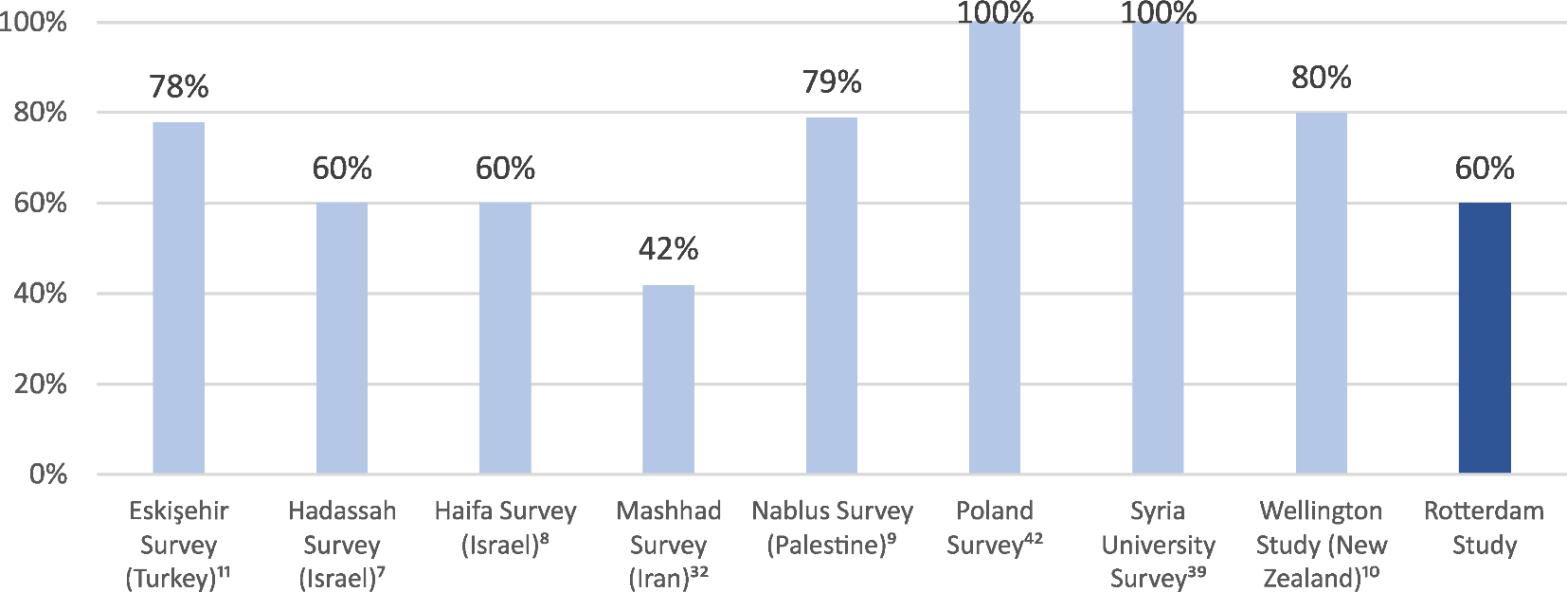
Unawareness rates reported in different screening studies.

One thought-provoking finding of our study is that 6 out of 10 keratoconus patients were unaware of their condition or did not report it. Two-thirds of these patients had varying degrees of accompanying visual acuity loss. Causes that explained their low visual acuity were retrospectively investigated and included strabismus, amblyopia, glaucoma, and corneal trauma. It was not easy to establish the degree to which keratoconus might have contributed to their low vision. Previous screening studies have also shown high unawareness rates in developing as well as developed countries (Fig. 3)^7–11,32,39,42^. Such findings warrant further investigation about their cause. Understanding this can potentially be relevant from a public health standpoint and lead to improved referral or screening patterns. Notably, two unaware patients (aged 48 and 51) had a completely normal visual acuity, suggesting that immediate cross-linking upon diagnosis may not always be the best strategy.

### Strengths and limitations

Despite the relatively large size of this study, the limited number of manifest keratoconus cases in our cohort prevented us from establishing prevalence estimates with narrower confidence intervals. This challenge is inherent when studying a relatively rare disease in a population-based cohort. Additionally, the evidence-based Rotterdam Study definition was not validated in an external cohort. Similarly, the other compared definitions lacked external validation. Also, the few participants who used contact lenses (only 5 among the 72 probable cases) were instructed to stop wearing them only one night before the exam, which might not be ideal. To address this, we incorporated Holladay’s criteria in the definition^14^. Furthermore, screening studies like the Rotterdam Study might also experience a slight selection bias, as patients who regularly visit an ophthalmologist for chronic ocular diseases might be less motivated to undergo voluntary ophthalmic exams at a research centre. Finally, the ethnicity and risk factor analyses are reported in this study for the sake of completeness, but they are limited by the small number of cases. Given the exploratory nature of the analysis, unadjusted P-values were reported, however, multiple-testing correction might have been warranted. Moreover, risk factors were also measured long after the peak age of keratoconus incidence, so results should be interpreted with caution.

Strengths of this study include relying on a well-designed, well-conducted and relatively large population-based cohort. The proposed definition integrates various evidence-based diagnostic approaches for enhanced accuracy, and incorporates the three diagnostic recommendations of the global consensus on keratoconus and ectatic diseases^62^. The proposed definition demonstrated a substantial inter-observer agreement, highlighting its reliability. To the best of our knowledge, this is the first attempt to compare how various keratoconus definitions perform in a population-based cohort, providing critical insights into their relative performance, strengths and limitations. Supplementary Tables 2 and 3 provide a comprehensive overview of the published definitions, which can facilitate designing future screening studies. Finally, our study was one of very few population-based screening studies to assess prevalence in an older cohort. This group, which is older than the usual incidence age, facilitates approximating the lifetime risk of keratoconus. This is usually a challenge when screening young students or military conscripts.

In summary, our study calls for caution in reporting and comparing keratoconus prevalence figures from screening studies due to the large variability of the used diagnostic criteria. We show how relying on keratometry or BAD-D as standalone diagnostic criteria for keratoconus can have serious shortcomings. This highlights the importance of adopting a more nuanced and comprehensive definition, such as the one proposed in this paper. It also underlines the necessity of working towards a consensus that standardizes how keratoconus is defined in epidemiological studies.

## Electronic supplementary material

Below is the link to the electronic supplementary material.

Supplementary Material 1

## Data Availability

Data analysed or generated in this study can be made available upon reasonable request made to the corresponding author (subject to approval by the Rotterdam Study management team).
