# Supplementary material for: Different keratoconus definitions can lead to substantial prevalence disparities in population-based studies
Source: Sci Rep. 2025 Jan 28;15:3483. doi: 10.1038/s41598-025-87532-4 (PMC11775297; doi:10.1038/s41598-025-87532-4)
Supplement: Supplementary file 1 — Supplementary Material 1 [file 41598_2025_87532_MOESM1_ESM.pdf]

**SUPPLEMENTARY MATERIAL**

**Article Title:**

Keratoconus in a population-based study: how different definitions can lead to substantial prevalence disparities

**Authors:**

Hasan Shabani, MD, Bart T. H. van Dooren, MD, PhD, Magda A. Meester-Smoor, PhD, Annette A. J. M. Geerards, MD,

Caroline C. W. Klaver, MD, PhD, Wishal D. Ramdas, MD, PhD.

**INDEX**

**Page 2.** Supplementary Figure S1

**Page 3.** Supplementary Methods S1

**Page 3.** Supplementary Methods S2

**Page 4.** Supplementary Methods S3

**Page 6.** Supplementary Table S1

**Page 8.** Supplementary Table S2

**Page 13.** Supplementary Table S3

**Page 14.** Supplementary Table S4

**Page 15.** References for Supplementary Material

## SUPPLEMENTARY FIGURES

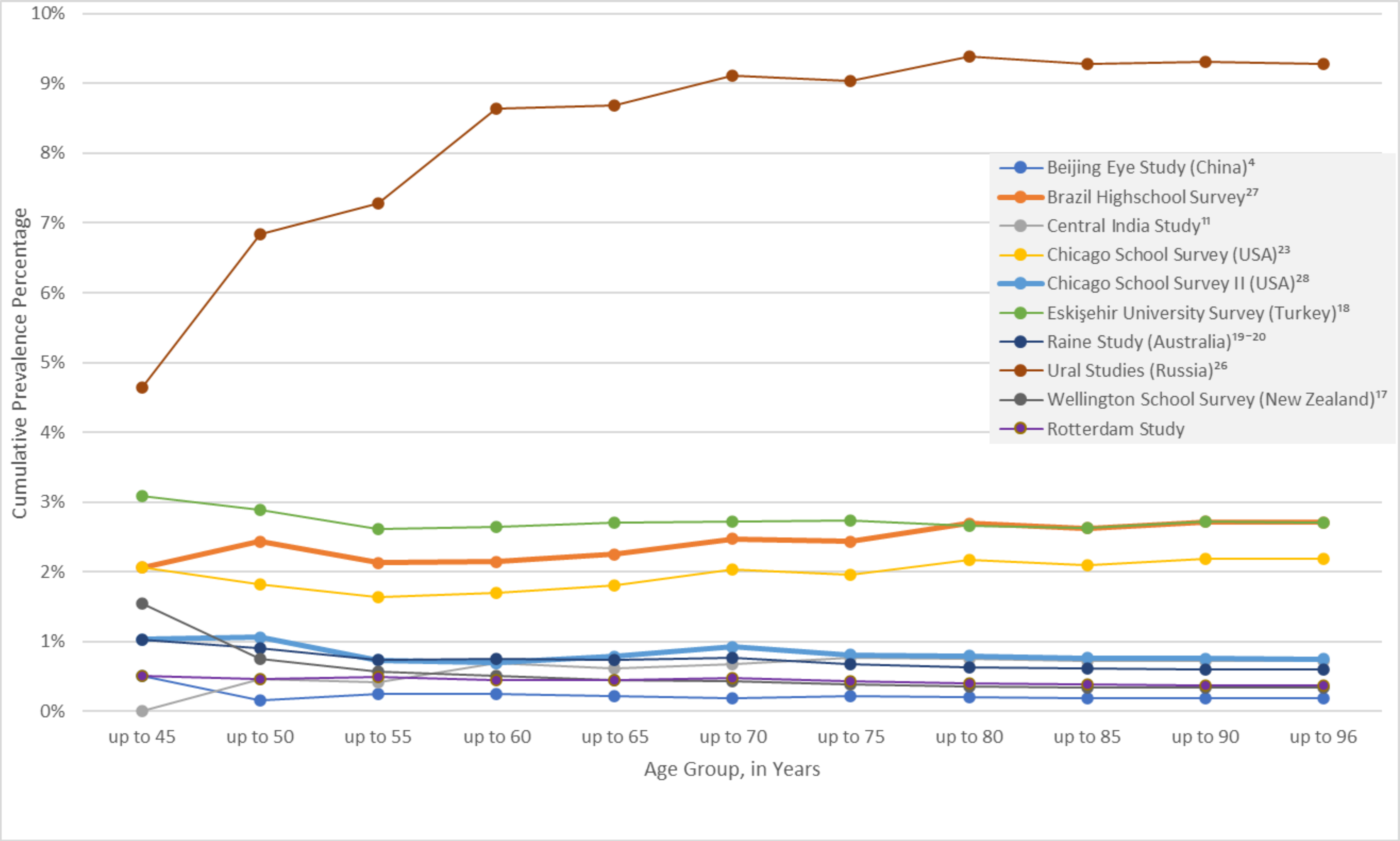


**Supplementary Figure S1. Cumulative prevalence percentage in The Rotterdam Study per age group according to each applied definition.** Eskişehir University Survey used two definitions. However, only the definition based on the Topographic Keratoconus index (TKC) was readily applicable to our dataset.

## SUPPLEMENTARY METHODS

### Supplementary Methods S1: Pentacam Workings and Capture Protocol

The Pentacam employs a rotating camera that captures images in three planes. It also relies on the Scheimpflug principle to generate a 3-dimensional model of the anterior eye segment. This model includes the anterior and posterior corneal surfaces, the iris and the crystalline lens, and gets generated from as many as 138000 independent elevation points. A secondary camera allows the detection of eye movements to correct for them as necessary. After each scan, many parameters, indices and maps are generated by the Pentacam software. This allows for a detailed assessment of corneal curvature, pachymetry, elevation, pachymetry distribution, astigmatism, sphericity, regularity, volume, densitometry and symmetry of the cornea[1]. When compared to other Scheimpflug imaging devices (e.g., Galilei and Sirius), Pentacam measurements were shown to have good repeatability[2].

Pentacam scans were captured by trained research assistants, and contact lens wearers were asked to take their lenses out at least one night before the exam. The scans were visualized using Pentacam software version 1.26r28 (Oculus Optikgeräte GmbH, Wetzlar, Germany), and the exports were done using Oculus Patient Data Management software version 6.10r59, from the same company. Scans that do not have an “OK” quality score as given by Pentacam’s quality score algorithm were excluded. Some participants had multiple reliable scans taken during the same session. This usually happens when the examiner is not satisfied with the quality of the first scan despite an “OK” score. Therefore, if multiple reliable scans were available, the last reliable scan was considered the most reliable and used in the analysis.

### Supplementary Methods S2: Literature Review to Find Alternative Definitions

To make an overview of the relevant publications and definitions, we relied partly on a recent comprehensive literature review from 2022[3]. Since multiple recent studies have also examined the prevalence of keratoconus after the publication of the aforementioned review, we also made a quick non-systematic literature review via PubMed and Scopus covering the period extending between 1 January 2021 and 20 October 2024 using the following key phrase (keratoconus AND prevalence). All abstracts were screened and filtered by one reviewer (H.S.). Next, the retrieved publications were examined by the same reviewer to assess their relevance. Studies done on specific patient groups (e.g., refractive surgery patients) were excluded. Reference lists of each eligible article were also screened to identify any additional relevant publications. After summarizing the relevant definitions, they were filtered once more to select the ones that are readily applicable to the Rotterdam Study. We selected those definitions that **(1)** relied on data from diagnostic instruments that are available or comparable to the Rotterdam Study, and **(2)** did not necessitate qualitative assessment (i.e., map patterns' recognition) of ≥100 scans, as this was impractical. A total of fifteen population-based screening prevalence studies[4-18] were identified from the literature review of Santodominigo et al.[3], and twelve additional relevant studies were retrieved by searching PubMed and Scopus[19-30]. Scanning reference lists of the included articles did not yield any additional eligible studies. Supplementary Tables S1 and S2 summarize all the identified studies and the published definitions, including the ones that were readily applicable to The Rotterdam Study dataset.

**Supplementary Methods S3: Covariates and Risk Factor Assessment Methods**

Rotterdam Study participants were asked by trained research assistants whether they have regularly suffered from a frequently dry skin in the last year (yes/no), and whether they have ever suffered from an itchy skin disorder (yes/no). They were also asked whether they were ever diagnosed as having hay fever or dust mite allergy by a medical doctor (yes/no). Asthma cases were defined as participants with a physician's diagnosis of asthma reported

in their medical file[31]. Diabetes mellitus was defined in line with the World Health Organization criteria as fasting glucose ≥7.0 mmol/L, or non-fasting glucose >11.0 mmol/L[32]. Alternatively, having a prescription of insulin or oral antidiabetic medication was accepted as evidence of having diabetes. Participants were also asked whether they or their partners had noticed that they snored while asleep. For all the variables, data from the last interview was used. When this was not available, we used data from a previous interview. Awareness of having keratoconus or lack thereof was assessed by thoroughly examining the ocular history of each confirmed patient. Each suspected risk factor was assessed using multivariable logistic regression adjusted for age and sex. Next, we calculated for each potential risk factor the odds ratio (OR) with the corresponding 95% confidence interval (CI). The analysis was repeated for every compared definition.

**Supplementary Table S1** Comparison of keratoconus definitions used in other population-based screening studies

| | | Clinical signs | | | Corneal imaging | | |
|---|---|---|---|---|---|---|---|
| Year | Study | SL | RS | VA | Parameters | Indices | Map patterns |
| 2021 | Abu-Dhabi School Survey (UAE) [8] | - | - | - | ✓ | ✓ | ✓ |
| 2012 | Beijing Eye Study (China) [4] | - | - | - | ✓ | - | - |
| 2012 | Beirut Hospital Survey (Lebanon) [12] | - | - | - | - | ✓ | ✓ |
| 2024 | Brazil Highschool Study [27] | - | - | - | - | ✓ | - |
| 2009 | Central India Study [11] | - | - | - | ✓ | - | - |
| 2022 | Chicago School Survey I (USA) [23] | - | - | - | - | ✓ | - |
| 2024 | Chicago School Survey II (USA) [28] | - | - | - | ✓ | ✓ | - |
| 2024 | Eastern Saudi Arabia Study [29] | - | - | - | ✓ | ✓ | - |
| 2021 | Eskişehir University Survey (Turkey) [18] | - | - | - | ✓ | ✓ | ✓ |
| 1995 | French Army Survey [10] | - | - | - | - | ✓ | ✓ |
| 2023 | Gutenberg Health Study (Germany) [24] | - | - | - | ✓ | ✓ | ✓ |
| 2011 | Hadassah College Survey (Israel) [14] | - | - | - | ✓ | ✓ | ✓ |
| 2014 | Haifa College Survey (Israel) [15] | - | - | - | ✓ | ✓ | ✓ |
| 2021 | Israeli Military Survey [21] | - | ✓ | ✓ | ✓ | ✓ | - |
| 2014 | Mashhad University Survey (Iran) [7] | ✓ | ✓ | ✓ | ✓ | ✓ | ✓ |
| 2015 | Nablus University Survey (Palestine) [16] | - | - | - | ✓ | ✓ | ✓ |
| 2023 | Poland Primary Care Survey [25] | - | - | - | ✓ | ✓ | ✓ |
| 2021/2023 | Raine Study (Australia) [19,20] | - | - | - | - | ✓ | ✓ |
| 2018 | Riyadh Pediatric Survey (Saudi Arabia) [13] | - | - | - | ✓ | ✓ | ✓ |
| 2023 | Rotterdam Study (The Netherlands) | - | - | - | ✓ | ✓ | ✓ |
| 2013 | Shahroud Eye Study (Iran) [6] | - | - | - | ✓ | - | ✓ |
| 2023 | Shiraz University Study (Iran) [30] | ✓ | ✓ | - | ✓ | ✓ | - |

| | | | | | | | |
|---|---|---|---|---|---|---|---|
| 2022 | Syria University Survey [22] | ✓ | ✓ | - | - | ✓ | ✓ |
| 2013 | Tehran Eye Study (Iran) [5] | - | - | ✓ | ✓ | ✓ | ✓ |
| 1959 | United States Eye survey [9] | - | - | - | - | - | ✓ |
| 2023 | Ural Studies (Russia) [26] | - | - | - | ✓ | - | - |
| 2019 | Wellington KC Study (New Zealand) [17] | - | - | - | ✓ | ✓ | ✓ |

Corneal parameters represent a single corneal measurement, such as keratometry or pachymetry. Diagnostic indices, such as the BAD-D (final D of the Belin-Ambrosio enhanced ectasia Display) or the KPI (Keratoconus Percentage Index), are calculated from two or more corneal parameters.

SL, slit lamp signs including stromal thinning, Vocht's striae & Fleischer's ring; RS, retinoscopic scissoring; VA, visual acuity.

**Supplementary Table S2** Published keratoconus definition(s) from comparable population-based screening prevalence studies

| Study | Context | Notable exclusion criteria | Main diagnostic instrument(s) | Diagnostic criteria |
|---|---|---|---|---|
| **Abu-Dhabi School Survey (UAE - 2021)** [8] | Students from two secondary schools in Abu Dhabi | Subjects with a history of ocular surgery were excluded | Galilei G4 dual Scheimpflug and Placido imaging system (Ziemer Ophthalmic Systems AG, Port, Switzerland) | Clear tomographic patterns typical for keratoconus and ≥ 2 abnormal tomography score points were considered diagnostic.<br>Tomography scoring was done as follows: PPK> 20% (1 point), PPK> 45% (2 points), I-S> 1.4 D (1 point), I-S> 1.7 D (2 points), Kmax> 47.2 D (1 point), Pmin< 470 µm (1 point), AAI> 21.5 µm (1 point) |
| **Beijing Eye Study (China - 2012)** [4]* | The Beijing Eye Study | - | Lensstar 900H Low Coherence Reflectometry | A steep cornea/keratoconus was defined as a corneal refractive power of equal to or higher than 48 diopters in the steepest corneal meridian (using a refractive index of 1.3315) |
| **Beirut Hospital Survey (Lebanon - 2012)** [12] | Randomly selected medical students or residents in Beirut | - | Tomey Topographic Modelling System (TMS-4, Tomey Corp, Nagoya, Japan) | A positive assessment according to two composite indices (KCI and KSI)<br>**AND/OR**<br>a positive assessment of the topographic patterns by an ophthalmologist |
| **Brazil Highschool Study (2024)** [27]* | Seven high schools in the Greater Porto Alegre metropolitan region in Southern Brazil | - | Pentacam Scheimpflug tomography | Manifest keratoconus was diagnosed using a Belin/Ambrosio Enhanced Ectasia display III (BAD-D) cutoff of 2.6. At least one eye should meet the definition for a participant to classify as having manifest keratoconus. |
| **Central India Study (2009)** [11]* | Participants of the Central India Eye and Medical Study (in Maharashtra) | - | Keratometry was assessed using a non-automatic keratometer (Appassawamy Assoc, Chennai, India) | Keratoconus was defined as an anterior corneal refractive power exceeding 48 diopters, using a refractive index of 1.3375 |
| **Chicago School Survey I (USA - 2022)** [23]* | Children aged 3-18 years at a school-based vision clinic in Chicago | - | Pentacam Scheimpflug tomography | Belin/Ambrósio enhanced ectasia display score (BAD-D) of 2.7 or more in either eye |
| **Chicago School Survey II** | Children aged 3-18 years at a school-based vision clinic in Chicago | - | Pentacam Scheimpflug tomography | Keratoconus was established when having a Belin/Ambrósio enhanced ectasia display score (BAD-D) of ≥3.00 in addition to a back elevation at thinnest point (BETP) ≥18 mm for myopia or ≥28 mm for hyperopia/mixed astigmatism in at least one eye. |

| | | | | |
|---|---|---|---|---|
| **(USA - 2024)** [28]⋆ | | | | |
| **Eastern Saudi Arabia Study (2024)** [29] | Subjects between 13 and 23 years old were recruited from the hospital's patient population. | Subjects with a history of KCN, refractive surgery, any other corneal surgery, or any contact lens use except for occasional use. | Pentacam Scheimpflug tomography | An experienced corneal surgeon assessed the images, employing objective criteria such as keratometry values, maximum keratometry, thinnest corneal pachymetry, and Belin Ambrosio Enhanced Ectasia Display total D values to identify KCN patients or suspects. |
| **Eskişehir University Survey (Turkey - 2021)** [18]**†** | Randomly selected students and faculty members (aged ≥18 and ≤30 years) at the Medical and Health Sciences faculties of Eskişehir Osmangazi University | Subjects with a history of refractive surgery were excluded | Pentacam Scheimpflug tomography | Two definitions were used: [b]<br>**(1)** Eyes that had steepening on the tangential map, thinning on the relative pachymetry map, and elevation of more than 10 μm on the posterior elevation map on the Holladay 6 map display were classified as keratoconus.<br>**AND/OR**<br>**(2)** having a TKC≥ 1 |
| **French Army Survey (1995)** [10] | Young male subjects (French army conscripts) | - | Tomey Topographic Modelling System (TMS-1) | KCI index> 30%, in addition to a power distribution and map patterns compatible with the diagnosis of keratoconus. |
| **GHS Study (Germany, 2023)** [24] | Participants of the GHS population-based study | - | Pentacam Scheimpflug tomography | masked double grading by two experienced ophthalmologists. In case of different findings, a third ophthalmologist was consulted. |
| **Hadassah College Survey (Israel - 2011)** [14] | Students of Hadassah College (Jerusalem) | - Students who had refractive or corneal surgery (except for keratoconus management)<br>- students diagnosed with another ocular disease<br>- hard contact lens users | Tomey Topographic Modeling System (TMS-4) OR a CT-1000 corneal topographer (*Shin-Nippon*, Rexxam, Japan) | KISA%> 100% and both KCI and KSI> 0%; clear topographic pattern; corneal apex> 50D and/or I-S> 3.5D |
| **Haifa College Survey (Israel - 2014)** [15] | Arab volunteer students from the Academic Arab College of Education, in Haifa | Subjects with contact lens warpage, who had undergone refractive or corneal surgery (except for keratoconus management), who had been diagnosed with an ocular disease other than keratoconus and who worm hard contact lenses | Tomey Topographic Modelling System (TMS-4) | An eye was defined as manifest keratoconus if it fulfilled the following: KSI> 0%; KPI> 0%; clear topographic pattern; corneal apex> 50D and/or I-S> 3 |

| | | | | |
|---|---|---|---|---|
| **Israeli Military Survey (2021)** [21] | Nationwide survey of military recruits in Israel | - | Unspecified Topographer | **(1)** High suspicion based on history taking/visual acuity assessment, in addition to **(2)** central keratometry≥ 48 D, and **(3)** I-S≥ 1.4 |
| **Mashhad University Survey (Iran - 2014)** [7] | Students at Mashhad University of Medical Sciences | A positive history of refractive surgery or any ocular surgery or a positive history of using contact lenses for reasons other than keratoconus | Retinoscopy, Slit-lamp examination, Tomey TMS-4 and Orbscan-II Topographer (Bausch & Lomb Surgical, Salt Lake City, Utah, USA) | Diagnosis by ophthalmologist: based on the presence of suspicious topographic map patterns alongside indicative clinical findings (scissoring, Vogt's striae, Fleisher's ring, corneal thinning or Bowman's membrane rupture) with or without abnormal topography indices |
| **Nablus University Survey (Palestine - 2015)** [16] | Randomly selected students attending An-Najah National University (Nablus) | History of corneal pathology other than keratoconus, traumatic corneal scars, or history of corneal keratoplasty for reasons other than keratoconus | Pentacam Scheimpflug tomography | **(a)** The presence of significant abnormal pattern of elevation on anterior and/or posterior map; **AND (b)** At least six of the following features: (1) One of the asymmetrical patterns on sagittal map, (2) I-S value≥ 1.5 on the anterior curvature map at the 4mm circle, (3) Y-coordinate of the thinnest location≤ -1, (4) Superior - inferior difference on corneal thickness map> 30, (5) Anterior elevation map within the 4mm circle> 15, (6) Posterior elevation map within the 4mm circle> 20, (7) Abnormal (red) Indices of irregularity (8) Abnormal (red) Belin/Ambrσsio Enhanced Ectasia Display |
| **Poland Primary Care Survey (2023)** [25] | All patients aged 10–80 years subscribed to a single general practitioner in the Elbląg area, Poland | - | Scansys Anterior Segment Analyzer TA 517 (MediWorks Precision Instruments, Shanghai, China | Presence of tomographical map patterns for keratoconus (round, oval, superior steep, inferior steep, irregular, inferior-steep asymmetric bow tie, superior-steep asymmetric bow tie, and symmetric or asymmetric bow tie with skewed radial axis [SRAX] >21 degrees), accompanied by focal steepening (front elevation >7 μm and/or back elevation >17 μm) and corresponding corneal thinning, 3-mm inferior–superior (I-S) kerato-metric asymmetry >1.4 D or central Kmean >47.2 D. |
| **Raine Study (Australia - 2021)** [19]* | 20 years old participants of The Raine Study (Perth) | Participants with a history of ocular surgery or any disorder that may have affected imaging were excluded | Pentacam Scheimpflug tomography | **(1)** Belin/Ambrósio enhanced ectasia display score (BAD-D) of 2.6 or more in either eye, and **(2)** a confirming qualitative analysis by 2 cornea specialists to exclude false-positive scans |
| **Raine Study (Australia - 2023)** [20]* | 28 years old participants of The Raine Study (Perth) | - | Pentacam Scheimpflug tomography | **(1)** Belin/Ambrósio enhanced ectasia display score (BAD-D) of 2.6 or more in either eye, and **(2)** a confirming qualitative analysis by 2 cornea specialists to exclude false-positive scans |

| | | | | |
|---|---|---|---|---|
| **Riyadh Pediatric Survey (Saudi Arabia - 2018)** [13] | Saudi paediatric patients from 6-21 years of age who were seen at multiple non-ophthalmic emergency departments in Riyadh (KSA) | All patients with pre-existing ocular disease other than corneal ectasia or history of ocular surgery were excluded | Pentacam Scheimpflug tomography | Cases were independently assessed by two cornea specialists. Diagnosis was based on **(1)** subjective pattern analysis (focal abnormality of anterior corneal curvature with concomitant corneal thinning) combined with **(2)** objective metrics (maximum keratometry, regional corneal thickness values and Belin/Ambrósio Total D value (BAD-D) scores, among others) |
| **Shahroud Eye Study (Iran -2013)** [6] | Shahroud Eye Study | - | Pentacam Scheimpflug Tomography | Diagnosis was mainly based on Holladay's topographic criteria: **(1)** Apex of the cone is not centered at the 6 o'clock semi meridian; **(2)** the cone should appear round on the tangential map; **(3)** keratometry 45.00 diopters (D); **(4)** corneal thickness at the apex of the cone is approximately 30 mm thinner than the corresponding distance above the pupil center; and **(5)** topographic patterns are not symmetric. In addition to Holladay's criteria: **(6)** no history of ocular trauma leading to hospitalization and no corneal opacity or vascularization on slit-lamp examination |
| **Shiraz University Study (Iran)** [30] | Shiraz University of Medical Science employees, aged 21 to 62 years, were recruited. | - | Pentacam Scheimpflug Tomography | The final definition of KCN was established when there were clinical signs (scissor reflex and irregular astigmatism in retinoscopy or the signs of Vogt's striae, Fleischer rings, and Munson's sign in bio-microscopy) and two or more abnormal Pentacam parameters (Zonal Kmax-3 mm > 48 D, ART-max< 339, IS-value> 1.4, BAD-D> 1.6 |
| **Syria University Survey (2022)** [22] | Students from Tishreen University (Lattakia) and Damascus University | Participants with non-ectatic corneal pathology and/or a history of refractive surgery | Autorefractor keratometry (SEIKO CO, GR-3500KA, Japan), slit-lamp examination, dilated retinoscopy and corneal imaging with Sirius (Costruzione Strumenti Oftalmici, Florence, Italy) | The diagnosis of keratoconus was made if there was **(1)** an irregular cornea determined by distorted keratometry mires or/and distortion of the retinoscopic reflex, or one of the following slit-lamp findings: Vogt striae, 2-mm arc of Fleisher ring, or corneal scarring consistent with keratoconus, in addition to **(2)** a positive Sirius software keratoconus indicator |

| | | | | |
|---|---|---|---|---|
| **Tehran Eye Study (Iran - 2013)** [5] | Tehran Eye Study | - | Orbscan-II Topographer | Meeting at least four of the following five criteria: **(1)** Keratometry> 47.2 D; **(2)** ratio of anterior/posterior corneal curvature> 1.27; **(3)** thinnest point of cornea< 470 um; **(4)** posterior best-fit sphere> 52 D; **(5)** visual acuity worse than 20/40 in the chosen eye (the better eye if bilateral and the affected eye if unilateral) |
| **United States Eye survey (1959)** [9] | Keratoscopic survey of 13395 eyes of rural participants in the US | - | Placido disk-based Topography | Keratoconic patterns on Placido disk-based topography |
| **Ural Eye Studies (Russia - 2023)** [26]* | Participants of the Ural Eye Studies | - | Pentacam Scheimpflug tomography | Keratometry equal to or greater than 48 Diopters |
| **Wellington Keratoconus Study (New Zealand - 2019)** [17]* | A population-based cohort study involving year 9 and year 11 students in Wellington, NZ | - | Pentacam Scheimpflug tomography | **(1)** A BAD-D value greater than 1.88; alongside **(2)** a Topometric Keratoconus Classification (TKC) categorized as keratoconus; and **(3)** a pathologic Index of Height Decentration (IHD); and **(4)** a tomographic 4-map pattern analysis consistent with keratoconus |

* Definitions from these publications were readily applicable to our dataset. † Only definition (2) was readily applicable to our dataset.
PPK, percent probability of keratoconus; I-S, Inferior-Superior index; D, diopters; Kmax, maximum Keratometry; Pmin, minimum pachymetry; AAI, asphericity asymmetry index; KCI, Klyce/Maeda Keratoconus Index; KSI, Smoke/Klyce Keratoconus Severity Index; TKC, Topographic Keratoconus Classification; KISA%, a diagnostic keratoconus index proposed by Rabinowitz/Rasheed; KPI, Keratoconus Prediction Index; µm, microns; Zonal Kmax-3 mm: zonal maximum keratometry in a 3-mm zone around the steepest point.

**Supplemental Table S3** Multiple regression outcomes in the Rotterdam Study dataset according to each tested definition

| | Missing values<br>Number (%) | Beijing Eye Study (China)[4]<br>OR (95%CI) | Brazil Highschool Survey[27] | Central India Study[11]<br>OR (95%CI) | Chicago School Survey I (USA)[23]<br>OR (95%CI) | Chicago School Survey II (USA)[28]<br>OR (95%CI) | Eskisehir University Survey (Turkey)[18]<br>OR (95%CI) | Raine Study (Australia)[19,20]<br>OR (95%CI) | Ural Studies (Russia)[26]<br>OR (95%CI) | Wellington School Survey (New Zealand)[17]<br>OR (95%CI) | Rotterdam Study<br>OR (95%CI) |
|---|---|---|---|---|---|---|---|---|---|---|---|
| **Dry skin** | 22 (0.8) | * | 1.15 (0.71 - 1.87) | 6.14 (1.76 - 21.42)†† | 1.27 (0.74 - 2.18) | 1.32 (0.55 - 3.20) | 1.47 (0.91 - 2.38) | 1.66 (0.60 - 4.55) | 1.27 (0.97 - 1.67) | 6.50 (1.31 - 32.10)† | 7.48 (1.55 - 35.97)† |
| **Itchy skin** | 36 (1.4) | 0.84 (0.14 - 5.08) | 0.84 (0.51 - 1.37) | 0.75 (0.29 - 1.91) | 0.92 (0.53 - 1.60) | 0.83 (0.34 - 2.01) | 1.06 (0.66 - 1.70) | 1.02 (0.38 - 2.76) | 0.98 (0.75 - 1.28) | 0.62 (0.15 - 2.51) | 1.36 (0.39 - 4.75) |
| **Asthma** | 0 (0) | 2.18 (0.24 - 19.83) | 1.13 (0.54 - 2.40) | 0.94 (0.21 - 4.10) | 1.04 (0.44 - 2.44) | 1.56 (0.45 - 5.35) | 1.10 (0.52 - 2.32) | 1.39 (0.31 - 6.22) | 1.13 (0.74 - 1.70) | 1.26 (0.16 - 10.20) | 2.57 (0.54 - 12.28) |
| **Dust mite allergy** | 39 (1.5) | 1.55 (0.17 - 14.19) | 0.21 (0.05 - 0.85)† | 0.82 (0.19 - 3.60) | 0.26 (0.06 - 1.08) | 0.33 (0.04 - 2.49) | 0.74 (0.34 - 1.64) | 0.39 (0.05 - 3.00) | 0.90 (0.59 - 1.37) | 0.66 (0.08 - 5.34) | 0.69 (0.09 - 5.50) |
| **Hay fever** | 40 (1.5) | 2.88 (0.47 - 17.66) | 1.12 (0.61 - 2.08) | 2.74 (1.06 - 7.09)† | 1.34 (0.70 - 2.56) | 1.09 (0.36 - 3.30) | 1.37 (0.77 - 2.43) | 1.39 (0.44 - 4.40) | 1.02 (0.72 - 1.45) | 1.05 (0.21 - 5.13) | 1.89 (0.48 - 7.51) |
| **Diabetes** | 0 (0) | * | 1.05 (0.54 - 2.04) | 0.76 (0.17 - 3.36) | 1.08 (0.52 - 2.24) | 1.60 (0.52 - 4.90) | 1.06 (0.53 - 2.11) | 0.53 (0.07 - 4.10) | 1.10 (0.76 - 1.62) | 1.31 (0.16 - 10.95) | 0.82 (0.10 - 6.76) |
| **Ever Smoking** | 65 (2.4) | 1.12 (0.18 - 6.77) | 0.69 (0.43 - 1.12) | 1.58 (0.60 - 4.20) | 0.57 (0.33 - 0.97) | 0.56 (0.23 - 1.37) | 0.81 (0.50 - 1.31) | 0.73 (0.27 - 1.95) | 1.14 (0.86 - 1.50) | 0.62 (0.17 - 2.34) | 1.05 (0.29 - 3.78) |
| **Snoring at night** | 733 (27.6) | 1.85 (0.19 - 18.39) | 1.13 (0.62 - 2.05) | 2.02 (0.55 - 7.45) | 1.25 (0.64 - 2.44) | 1.26 (0.43 - 3.69) | 1.20 (0.65 - 2.20) | 1.31 (0.40 - 4.35) | 0.99 (0.72 - 1.36) | 1.74 (0.34 - 8.85) | 1.61 (0.32 - 8.14) |

* These models did not properly converge due to the low number of cases.

† Statistically significant at a p-value of 0.05. †† Statistically significant at a p-value of 0.005.

OR, odds ratio; CI, confidence interval.

Blue indicates OR > 1 while orange indicates OR < 1. Darker color accents indicate statistical significance at 0.05.

| **Supplementary Table S4.** Best Corrected Visual Acuity (BCVA) of Keratoconus Patients | | | |
|---|---|---|---|
| **Patient Number** | **BCVA OD** | **BCVA OS** | **Reported Having Keratoconus?** |
| 1 | 1.00 | 1.00 | No |
| 2 | 0.05 | 1.00 | No |
| 3 | 0.80 | 1.00 | No |
| 4 | 1.00 | 1.00 | No |
| 5 | 0.63 | 0.50 | No |
| 6 | 0.50 | 1.00 | No |
| 7 | 0.80 | 0.40 | Yes |
| 8 | 0.63 | 0.63 | Yes |
| 9 | 1.00 | 0.003 | Yes |
| 10 | ≥0.63* | ≥0.63* | Yes |

* The exact visual acuity data for this patient was not available.

**REFERENCES FOR SUPPLEMENTARY MATERIAL**


1 Oculus. *Measurement Principle, Licences & Network*, https://www.pentacam.com/int/technology/measurement-principle-licences-network.html (2023).

2 Shetty, R. *et al*. Repeatability and agreement of three scheimpflug-based imaging systems for measuring anterior segment parameters in Keratoconus. *Invest. Ophthalmol. Vis. Sci.* **55**, 5263-5268 (2014).

3 Santodomingo-Rubido, J. *et al*. Keratoconus: An updated review. *Cont Lens Anterior Eye* **45**, 101559 (2022).

4 Xu, L., Wang, Y. X., Guo, Y., You, Q. S. & Jonas, J. B. Prevalence and associations of steep cornea/keratoconus in greater beijing. the beijing eye study. *PLoS One* **7**, e39313-e39313; 10.1371/journal.pone.0039313 (2012).

5 Hashemi, H., Khabazkhoob, M. & Fotouhi, A. Topographic keratoconus is not rare in an Iranian population: The Tehran eye study. *Ophthalmic Epidemiol.* **20**, 385-391 (2013).

6 Hashemi, H. *et al*. Prevalence of Keratoconus in a Population-based Study in Shahroud. *Cornea* **32**, 1441-1445 (2013).

7 Hashemi, H. *et al*. The prevalence of keratoconus in a young population in Mashhad, Iran. *Ophthalmic Physiol. Opt.* **34**, 519-527 (2014).

8 Armstrong, B. K. *et al*. Screening for Keratoconus in a High-Risk Adolescent Population. *Ophthalmic Epidemiol.* **28**, 191-197 (2021).

9 Hofstetter, H. W. A keratoscopic survey of 13,395 eyes. *Optom. Vis. Sci.* **36**, 3-11 (1959).

10 Santiago, P. Y. *et al*. Prevalence of keratoconus and corneal topography in young male subjects. *Vision Res.* **36**, S307-S307 (1995).

11 Jonas, J. B., Nangia, V., Matin, A., Kulkarni, M. & Bhojwani, K. Prevalence and Associations of Keratoconus in Rural Maharashtra in Central India: The Central India Eye and Medical Study. *Am. J. Ophthalmol.* **148**, 760-765 (2009).

12 Waked, N., Fayad, A. M., Fadlallah, A. & El Rami, H. [Keratoconus screening in a Lebanese students' population]. *J. Fr. Ophtalmol.* **35**, 23-29 (2012).

13 Torres Netto, E. A. *et al*. Prevalence of keratoconus in paediatric patients in Riyadh, Saudi Arabia. *Br. J. Ophthalmol.* **102**, 1436-1441 (2018).

14 Millodot, M., Shneor, E., Albou, S., Atlani, E. & Gordon-Shaag, A. Prevalence and Associated Factors of Keratoconus in Jerusalem: A Cross-sectional Study. *Ophthalmic Epidemiol.* **18**, 91-97 (2011).

15 Shneor, E. *et al*. Prevalence of Keratoconus among Young Arab students in Israel. *International Journal of Keratoconus and Ectatic Corneal Diseases* **3**, 9-14 (2014).

16 Shehadeh, M. M. *et al*. Prevalence of Keratoconus Among a Palestinian Tertiary Student Population. *Open Ophthalmology Journal* **9**, 172-176 (2015).

17 Papali'i-Curtin, A. T. *et al*. Keratoconus Prevalence Among High School Students in New Zealand. *Cornea* **38**, 1382-1389 (2019).

18 Özalp, O., Atalay, E. & Yıldırım, N. Prevalence and risk factors for keratoconus in a university-based population in Turkey. *J. Cataract Refract. Surg.* **47**, 1524-1529 (2021).

19 Chan, E. *et al*. Prevalence of Keratoconus Based on Scheimpflug Imaging: The Raine Study. *Ophthalmology* **128**, 515-521 (2021).

20 Chan, E. *et al*. Incidence and Prevalence of Keratoconus Based on Scheimpflug Imaging. *Ophthalmology* **130**, 445-448 (2023).

21 Eliasi, E. *et al*. The Association Between Keratoconus and Body Mass Index: A Population-Based Cross-Sectional Study Among Half a Million Adolescents. *Am. J. Ophthalmol.* **224**, 200-206 (2021).

22 Salman, A. *et al*. Prevalence of Keratoconus in a Population-Based Study in Syria. *Journal Of Ophthalmology* **2022**, 6064533 (2022).

23 Block, S. S. *et al*. Prevalence of abnormal corneas in the United States based on Scheimpflug tomography analytics of a pediatric population. *Invest. Ophthalmol. Vis. Sci.* **63**, 2415 – A0218 (2022).

24 Marx-Gross, S. *et al*. Much higher prevalence of keratoconus than announced results of the Gutenberg Health Study (GHS). *Graefe's archive for clinical and experimental ophthalmology* **261**, 3241-3247 (2023).

25 Kanclerz, P., Przewłócka, K., Toprak, I. & Alio, J. The prevalence of keratoconus in northern Poland: A cross-sectional study of patients from a primary healthcare practice. *Cont. Lens Anterior Eye* **46**, 101846-101846 (2023).

26 Bikbov, M. M. *et al*. Prevalence and Associations of Keratoconus Among Children, Adults, and Elderly in the Population-Based Ural Eye Studies. *APJO* **12**, 591-603 (2023).

27 de Azevedo Magalhães, O., Pagano, B. N., Grellmann, L. V., Zago, V. S. & Kronbauer, C. L. Prevalence of Keratoconus Among High School Students in Southern Brazil: A Community-Based Study. *Eye Contact Lens* **50**, 117-120 (2024).

28 Harthan, J. S. *et al*. Prevalence of Keratoconus Based on Scheimpflug Corneal Tomography Metrics in a Pediatric Population From a Chicago-Based School Age Vision Clinic. *Eye Contact Lens* **50**, 121-125 (2024).

29 Khattak, A., Altalhi, A., Alotaibi, A. B. & Khattak, A. M. Prevalence of Keratoconus in the Young Eastern Population of Saudi Arabia. *Cureus* **16**, e55692; 10.7759/cureus.55692 (2024).

30 Mohaghegh, S. *et al*. Prevalence and risk factors of keratoconus (including oxidative stress biomarkers) in a cohort study of Shiraz university of medical science employees in Iran. *BMC Ophthalmol.* **23**, 188 (2023).

31 de Roos, E. W. *et al*. Asthma and its comorbidities in middle-aged and older adults; the Rotterdam Study. *Respir. Med.* **139**, 6-12 (2018).

32 World Health, O. Definition and diagnosis of diabetes mellitus and intermediate hyperglycemia: report of a WHO/IDF consultation. 1-50 (Geneva, 2006).
